# Investigations on annual spreading of viruses infecting cucurbit crops in Uttar Pradesh State, India

**DOI:** 10.1038/s41598-021-97232-4

**Published:** 2021-09-09

**Authors:** Shweta Kumari, Nagendran Krishnan, Vikas Dubey, Bappa Das, Koshlendra Kumar Pandey, Jagdish Singh

**Affiliations:** 1 ICAR-Indian Institute of Vegetable Research, Varanasi, Uttar Pradesh 221305 India; 2 ICAR-Central Coastal Agricultural Research Institute, Old Goa, Goa 403402 India

**Keywords:** Pathogens, Virology

## Abstract

During 2018 an intensive study was conducted to determine the viruses associated with cucurbitaceous crops in nine agroclimatic zones of the state of Uttar Pradesh, India. Total of 563 samples collected and analysed across 14 different cucurbitaceous crops. The results showed the dominance of *Begomovirus* (93%) followed by *Potyvirus* (46%), cucumber green mottle mosaic virus (CGMMV-39%), *Polerovirus* (9%), cucumber mosaic virus (CMV-2%) and *Orthotospovirus* (2%). Nearly 65% of samples were co-infected with more than one virus. Additionally, host range expansion of CMV, CGMMV and polerovirus was also observed on cucurbit crops. A new potyvirus species, zucchini tigre mosaic virus, earlier not documented from India has also been identified on five crops during the study. Risk map generated using ArcGIS for virus disease incidence predicted the virus severity in unexplored areas. The distribution pattern of different cucurbit viruses throughout Uttar Pradesh will help identify the hot spots for viruses and will facilitate to devise efficient and eco-friendly integrated management strategies for the mitigation of viruses infecting cucurbit crops. Molecular diversity and evolutionary relationship of the virus isolates infecting cucurbits in Uttar Pradesh with previously reported strains were understood from the phylogenetic analysis. Diverse virus infections observed in the Eastern Plain zone, Central zone and North-Eastern Plain zone indicate an alarming situation for the cultivation of cucurbits in the foreseeable future.

## Introduction

Cucurbits are economically important vegetables grown extensively in tropical and subtropical regions for human consumption. The family Cucurbitaceae comprises 825 species in 118 genera^1^, of which about 100 species belonging to 37 genera are reported to be present in India. An area of nearly, 0.6 million hectares was dedicated to cucurbits cultivation with an annual production of 10.86 million tonnes in Uttar Pradesh during 2017–18^2^. Diseases caused by viruses are among the limiting factors to cucurbits cultivation worldwide. More than 70 different viruses were reported to cause a wide range of diseases in cucurbits^3^. To date, viruses in the genera *Begomovirus, Potyvirus, Cucumovirus, Tobamovirus, Tymovirus, Nepovirus*, and *Polerovirus* have been described as potential threats in cucurbits cultivation across the world^4–11^. Viruses belonging to these genera are mainly transmitted by insect-vectors, although seed transmission has been reported in some species belonging to the genera *Potyvirus, Cucumovirus*, and *Tobamovirus*^12–14^. Recently, six viruses associated with cucurbit crops have been documented from Southern India. They are *Papaya ring spot virus* (PRSV), *Cucumber green mottle mosaic virus* (CGMMV), *Zucchini yellow mosaic virus* (ZYMV), *Cucumber mosaic virus* (CMV) and *Begomovirus* (tomato leaf curl New Delhi virus and squash leaf curl China virus)^15^.

Studies on the occurrence and distribution of viruses associated with cucurbit crops have been done only in southern India. So far, little effort has been made to determine the viruses infecting cucurbitaceous crops in Northern India. Therefore, the objective of the present study was to perform a comprehensive analysis of the occurrence, distribution and genetic diversity of viruses infecting cucurbit crops across Uttar Pradesh, India. Furthermore, an attempt was also made to map the epidemiological distribution and incidence proportion of the afore-mentioned viruses associated with cucurbit crops.

## Results

### Symptomatology and dynamics of disease incidence in Uttar Pradesh

**Figure 1:**
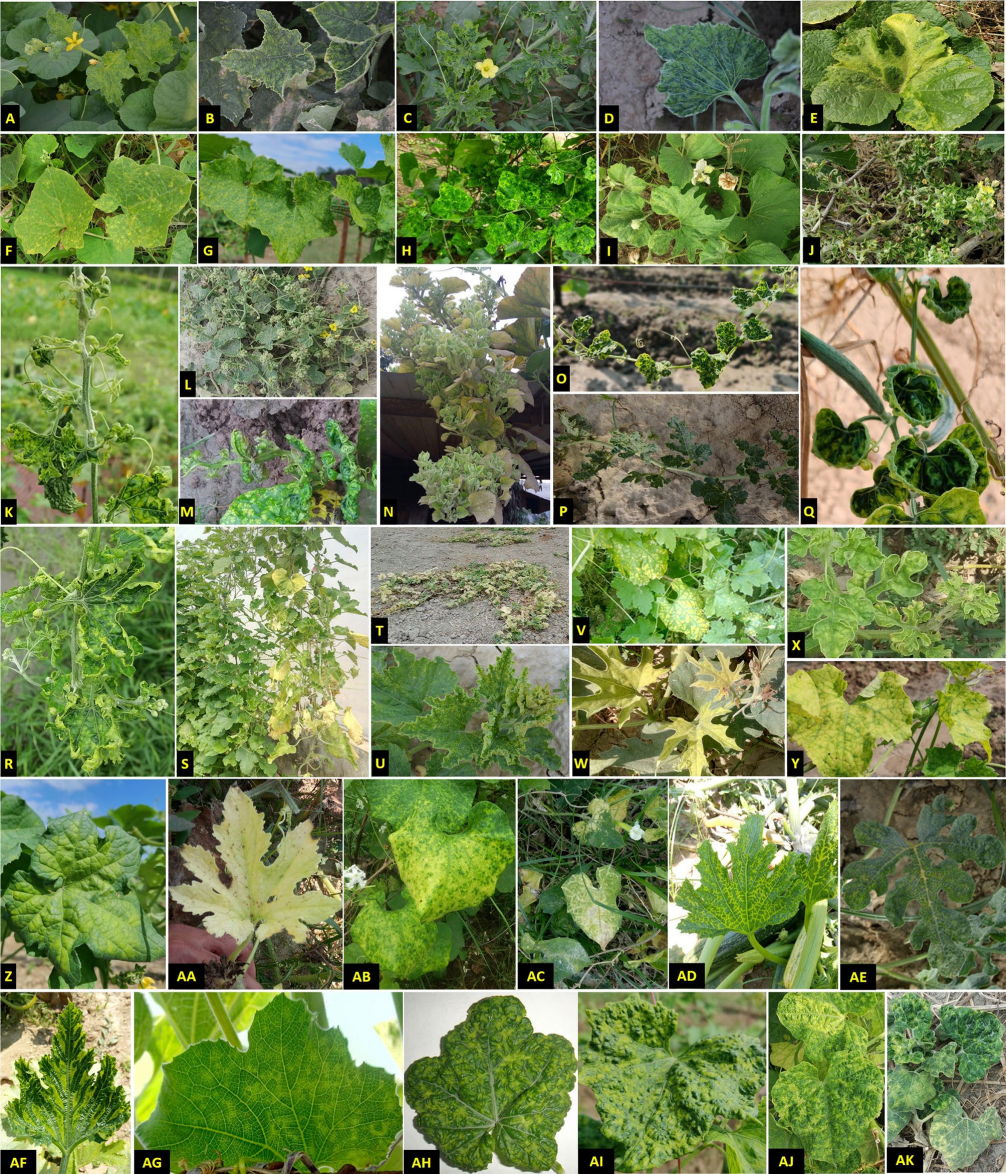
Symptoms of mosaic on Long melon (**A**), Cucumber (**B**), Bitter gourd (**C**), Bottle gourd (**D**), Pumpkin (**E**), Pointed gourd (**F**), Satputia (**G**), and Ivy gourd (**H**); leaf malformations on Bottle gourd (**I**), Bitter gourd (**J**,**K**), Cucumber (**L**), Sponge gourd (**M**), Bottle gourd (**N**), Satputia (**O**); leaf curl on Watermelon (**P**), Sponge gourd (**Q**), and Bitter gourd (**R**); chlorosis and yellowing on Musk melon (**S**), Pumpkin (**T**,**U**), Bitter gourd (**V**), Bottle gourd (**W**), Watermelon (**X**), Satputia (**Y**,**Z**), Ash gourd (**AA**), and Snake gourd (**AB**); clearing of veins in Ponited gourd (**AC**), Squash (**AD**) and Watermelon (**AE**); vein banding in Squash (**AF**); ring spots on Bottle gourd leaves (**AG**); and puckering and blistering of leaves on sponge gourd (**AH**), Satputia (**AI**), Ridge gourd (**AJ**) and Pumpkin (**AK**).

**Figure 2:**
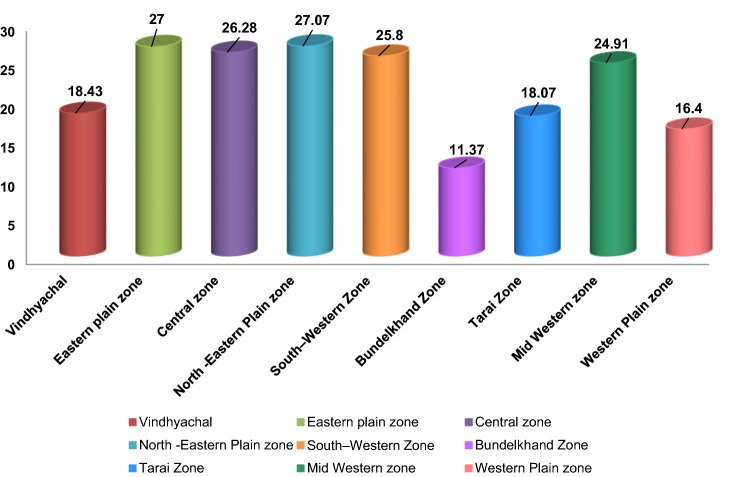
Viral disease incidence on cucurbits across the different agroclimatic zones of Uttar Pradesh.

A total of 563 samples from 14 different types of cucurbit crops were collected from nine agro-climatic zones of Uttar Pradesh (Supplementary Fig. 1). The samples showed virus like symptoms including mosaic, yellowing, mottling with chlorotic spots, distortion of leaves, puckering, yellowing, vein clearing, upward curling of leaves, necrosis, and stunting of plant growth with reduction in leaf size across farmers’ fields (Fig. 1). Virus incidence was found in all agro-climatic zone of Uttar Pradesh and the incidence and distribution of the infected cucurbits viruses were varied based on the region of the collecting areas (i.e., the greater the growing area, the more the diseased samples collected). These three zones viz., Eastern, central and north-eastern plain zone accounts for more than 50 percent of total diseased samples (Supplementary Fig. 2). Based on symptomatology average disease incidence varied between different agro-climatic zones ranging between 11 and 27% with an overall mean incidence of 24%. Our data revealed that the highest viral incidence was found in the Eastern and North-Eastern Plain zone (27%), followed by the central zone (26%) and the south-western zone (25%), respectively. The lowest disease incidence (11%) was recorded from the Bundelkhand zone (Fig. 2). Based on average field disease incidence, round melon (90%), satputia (72%), squash (56.07%) and watermelon (38.14%) were more prone to viral diseases (Table 1).

**Table 1:** Disease incidence of viruses in different cucurbit crops collected across Uttar Pradesh.

Crops	No. of present viruses	Disease incidence range (%)	Average disease incidence
Bottle gourd	POTYVIRUS, CGMMV, POLEROVIRUS, BEGOMOVIRUS	4–60	17.62
Sponge gourd	POTYVIRUS, CGMMV, CMV, POLEROVIRUS, BEGOMOVIRUS	7–60	30.14
Bitter gourd	POTYVIRUS, CGMMV, CMV, POLEROVIRUS, BEGOMOVIRUS	6–40	23.46
Ridge gourd	POTYVIRUS, CMV, BEGOMOVIRUS	0–10	10.00
Snake gourd	CGMMV, POTYVIRUS, BEGOMOVIRUS	5–13	9.33
Ivy gourd	POTYVIRUS, CGMMV, POLEROVIRUS, BEGOMOVIRUS	2–8	4.67
Pumpkin	POTYVIRUS, CGMMV, POLEROVIRUS, BEGOMOVIRUS	10–50	26.68
Squash	POTYVIRUS, CGMMV, POLEROVIRUS, BEGOMOVIRUS	30–89	56.07
Satputia	BEGOMOVIRUS	0–72	72.00
Cucumber	POTYVIRUS, CGMMV, BEGOMOVIRUS	2–25	11.52
Watermelon	POTYVIRUS, WBNV, CGMMV, BEGOMOVIRUS	14–90	38.14
Muskmelon	CGMMV, BEGOMOVIRUS	7–12	9.50
Long melon	POTYVIRUS, CGMMV, BEGOMOVIRUS	5–50	33.40
Round melon	WBNV	0–90	90.00

### Virus detection and analysis of distribution pattern

All samples collected during the study were subjected to PCR for Begomoviruses and RT-PCR for RNA viruses such as potyviruses (PRSV and ZYMV), *Cucumovirus* (CMV), *Tobamovirus* (CGMMV), *Orthotospovirus* (PBNV and WBNV), *Potexvirus*, *Crinivirus* and poleroviruses. Out of 563 collected cucurbits samples, 95.4% (537/563) were found to be infected with viruses (either single or mixed infection)*.* Despite showing virus like symptoms, the remaining 4.6% of samples tested negative for viruses. Universal and virus-specific primer pairs were used to test samples for eight virus genera, out of which samples were found to be positive for six genera (*Potyvirus, Cucumovirus, Tobamovirus, Polerovirus, Orthotospovirus* and *Begomovirus*). None of the samples were found to be positive for *Potexvirus* and *Crinivirus*. The relative frequency of viruses infecting cucurbit samples in our study showed dominance of *Begomovirus* (93%) followed by *Potyvirus* (46%), CGMMV (39%), poleroviruses (9%), CMV (2%) and *Orthotospovirus* (2%).

**Figure 3:**
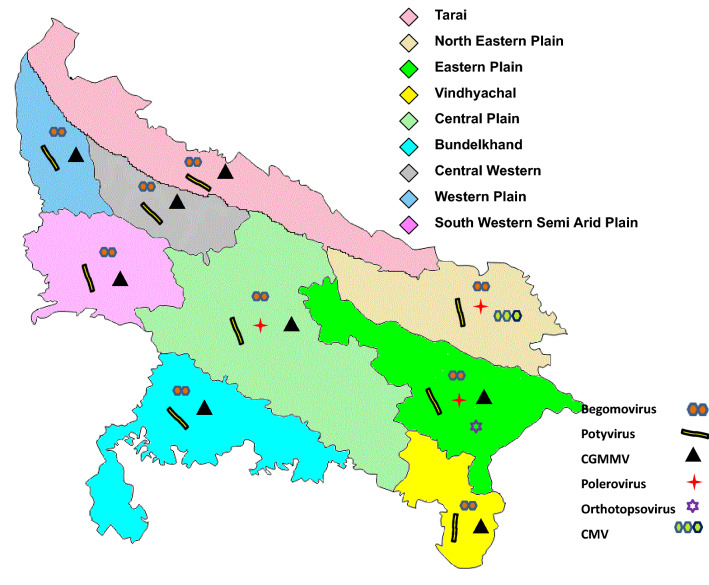
Map of agro-climatic zones of UP showing virus disease incidence and their virus distribution.

In general, the incidence of *Begomovirus* was higher in all the zones irrespective of the cucurbit crops. Diverse virus infection was observed only in samples tested from the Eastern Plain zone, Central zone and North-Eastern Plain zone while samples collected from the rest of the zones were positive for only potyviruses and CGMMV. Surprisingly, *Polerovirus* was primarily restricted to the Eastern plain zone, Central zone and North-Eastern Plain zone whereas *Orthotospovirus* and CMV were detected only among a few samples collected from the Eastern Plain zone and the North-Eastern Plain zone, respectively (Fig. 3). Potyvirus and CGMMV were the most frequent viruses detected among 64.3% samples associated with 12 different cucurbit crops (Supplementary Fig. 3).

### Expanded host range of viruses and new reports

Viruses detected in this study have already been documented and characterized from different parts of India. From this study, expansion of viral host ranges on different cucurbits was observed. Infection by poleroviruses such as cucurbit aphid borne yellow virus (CABYV) on squash; melon aphid borne yellow virus (MABYV) on ivy gourd; Luffa aphid borne yellow mosaic (LABYV) on sponge gourd, bitter gourd and pumpkin has been recorded for the first time from India. In addition, CMV on sponge gourd, ivy gourd and ridge gourd and CGMMV on long melon were also documented. Besides, Zucchini tigre mosaic virus (ZTMV) was observed newly in India on pumpkin, bottle gourd, bitter gourd, squash and cucumber.

### Mixed infection

**Figure 4:**
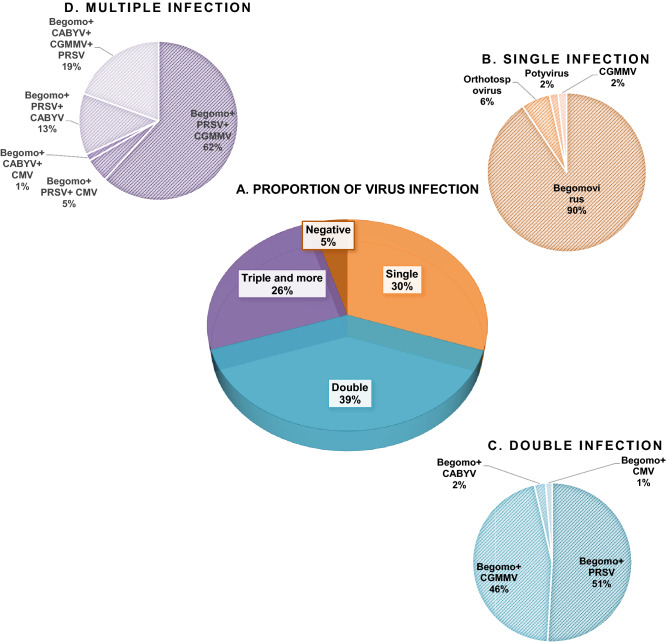
Status of mixed virus infection in cucurbits samples.

Virus-infected cucurbit samples showed a preponderance of mixed infection over single infection (Fig. 4). Among the tested samples, 366 samples (65%) were found to be co-infected with more than one virus (Fig. 4A). Among single infection (169 samples), infection dominated with *Begomovirus* (90%) followed by *Orthotospovirus* (6%), *Potyvirus* (2%) and CGMMV (2%) (Fig. 4B). Of the mixed infection, 61% of samples (223 samples) were doubly infected in combinations of Begomovirus + Potyvirus, Begomovirus + CGMMV, Begomovirus + Polerovirus and Begomovirus + CMV (Fig. 4C). Moreover, triple infections were also found in 31% of samples (114 samples) in four different combinations as *Begomovirus* + *Potyvirus* + CGMMV, *Begomovirus* + *Potyvirus* + CMV, *Begomovirus* + *Polerovirus* + CMV, *Begomovirus* + *Potyvirus* + *Polerovirus* and the remaining 8% of samples (29 samples) were infected with a combination of four viruses (*Begomovirus* + *Polerovirus* + CGMMV + *Potyvirus*) (Fig. 4D). Among mixed infections, *Potyvirus* and/or CGMMV in combination with *Begomovirus* was observed to be more dominant.

### Sequence analysis of viruses

In order to characterize the viruses infecting cucurbits in Uttar Pradesh state at the nucleotide level, selected samples were sequenced. Based on the sequencing, a CMV isolate infecting bitter gourd had 94.7% identity with the isolates reported earlier from Malaysia on cucumber (JN054637). The coat protein gene of the CGMMV isolate infecting cucurbits (bitter gourd, sponge gourd, cucumber, bottle gourd, long melon and snake gourd) was observed with more than 98% nucleotide identity with the CGMMV isolates recorded on various cucurbit crops characterised earlier from India. BLASTn analysis of nucleotide sequences amplified using a universal *Polerovirus* primer pair showed that three *Polerovirus* species (CABYV, MABYV and LABYV) were associated with different cucurbits of Uttar Pradesh. Similarly, sequence analysis using the BLASTn programme of the Nib region showed association of three potyvirus species (PRSV, ZYMV and ZTMV). In the coat protein region, PRSV showed more than 85% identity with isolates reported from India (EU475877) and China (KY933061); ZYMV showed > 98% identity with a *Cucurbita pepo* isolate (JN183062) reported from Iran; and ZTMV sequences showed > 85% identity with the sequences reported from France (KC345605, KC345607-8). Watermelon bud necrosis virus (WBNV) was the only *Orthotospovirus* found associated with both round melon and watermelon having identity of > 95% with Indian isolates (Table 2).

**Table 2:** List of isolates used in this study.

Virus	Isolate	Crop	GenBank Acc. no	Percent identity in BLAST with reference isolate	Reference isolate
CMV	UPCMV BG	Bitter gourd	MT768062	94.69%	JN054637
CGMMV	UPCGMMV BOG1	Bottle gourd	MT636375	98.72–99.09	MH271435
	UPCGMMV BOG2		MT636376		
	UPCGMMVLM	Long melon	MT636380	99.46–99.64	MH271435
	UPCGMMVCU	Cucumber	MT636384		
	UPCGMMVBG	Bitter gourd	MT636381	98.93–99.08%	DQ767631
	UPCGMMVSPG1	Sponge gourd	MT636382		
	UPCGMMV BOG3	Bottle gourd	MT636377	99.64	MH271409
	UPCGMMVSPG2	Sponge gourd	MT636383	96.05–99.09	MF510469
	UPCGMMVSNG	Snake gourd	MT636385		
	UPCGMMV BOG4	Bottle gourd	MT636378		
	UPCGMMV BOG5	Bottle gourd	MT636379		
PRSV	UPPRSV SPG1	Sponge gourd	MT636386	86.56	KT895257
	UPPRSV SPG2	Sponge gourd	MT636387	84.48	MN203187
	UPPRSV PUM1	Pumpkin	MT648432	89.61	EU475877
	UPPRSV SNG1	Snake gourd	MT636389	92.02–90.59	KP743981
	UPPRSV SNG2		MT636390		
	UPPRSV PUM	Pumpkin	MT636388	85.82	KF002603
	UPPRSV SNG	Snake gourd	MT648434	85.03–85.07	KY933061
	UPPRSV PUM2	Pumpkin	MT648433		
ZYMV	UPZYMV SPG1	Sponge gourd	MT636391	98.64–98.87	JN183062
	UPZYMV CU1	Cucumber	MT636392		
	UPZYMV SPG2	Sponge gourd	MT636393		
ZTMV	UPZTMV BOG1	Bottle gourd	MT755615	86.41	KC345607
	UPZTMV BOG2	Bottle gourd	MT755616	87.03	KC345608
	UPZTMV CU	Cucumber	MT755617	85.56	KC345607
	UPZTMV BG	Bittergourd	MT755618	87.80	KC345605
	UPZTMV PUM	Pumpkin	MT755614	87.46	MK988416
	UPZTMV SQ	Squash	MT755613	86.02	KC345605
LABYV	UPPOL BG1	Bitter gourd	MT622665	82.56	KR476808
	UPPOL BG2		MT622666		
	UPPOL PUM1	Pumpkin	MT622671	84.63	KR476808
	UPPOL PUM2	Pumpkin	MT622672	84.73	KF427702
	UPPOL SPG1	Sponge gourd	MT622667	83.72	KF427702
MABYV	UPPOL SQ1	Squash	MT622669	93.06	JQ700307
	UPPOL SQ2	Squash	MT622670	93.44	JQ700307
	UPPOL IVY1	Ivy gourd	MT622673	93.63	JQ700307
WBNV	VNS WM1	Watermelon	MN462627	95.53	EU373762
	VNS RM1	Round melon	MH717082	95.41	EU373763

### Phylogenetic analysis of viruses

**Figure 5:**
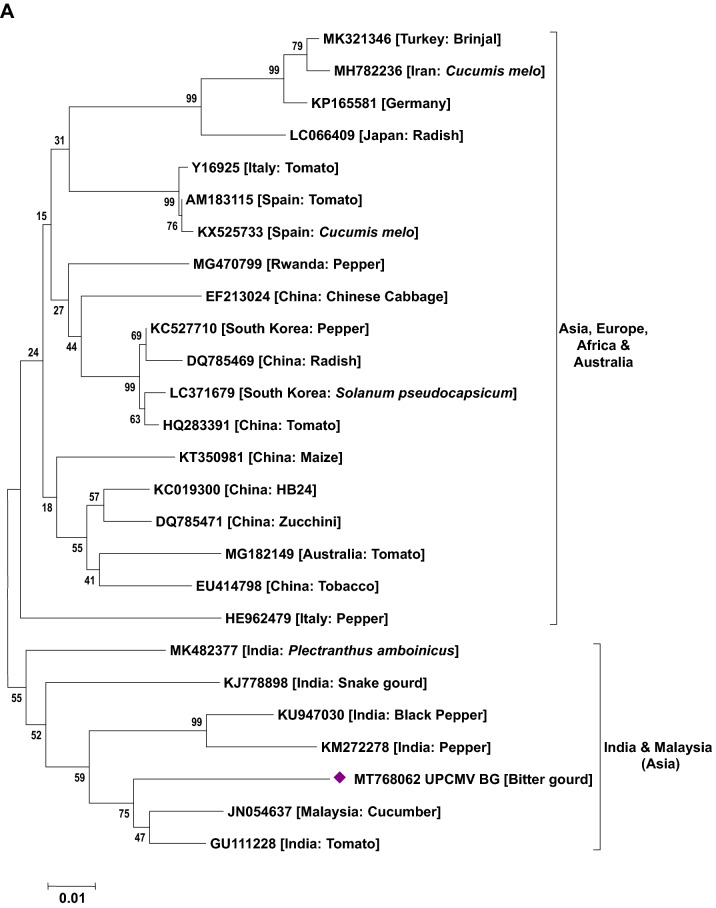

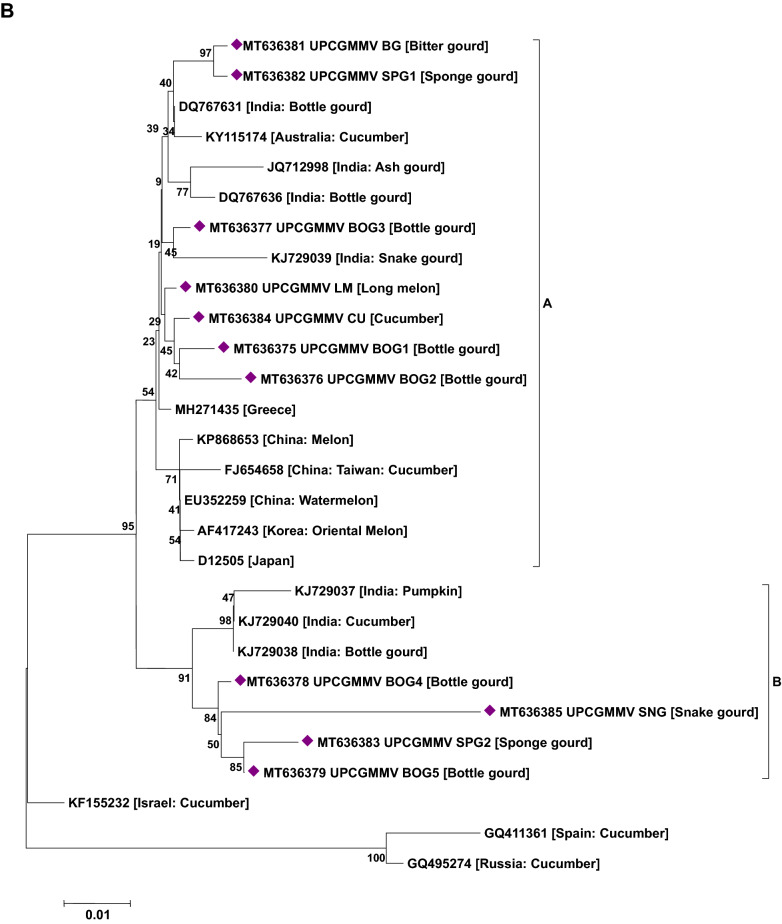

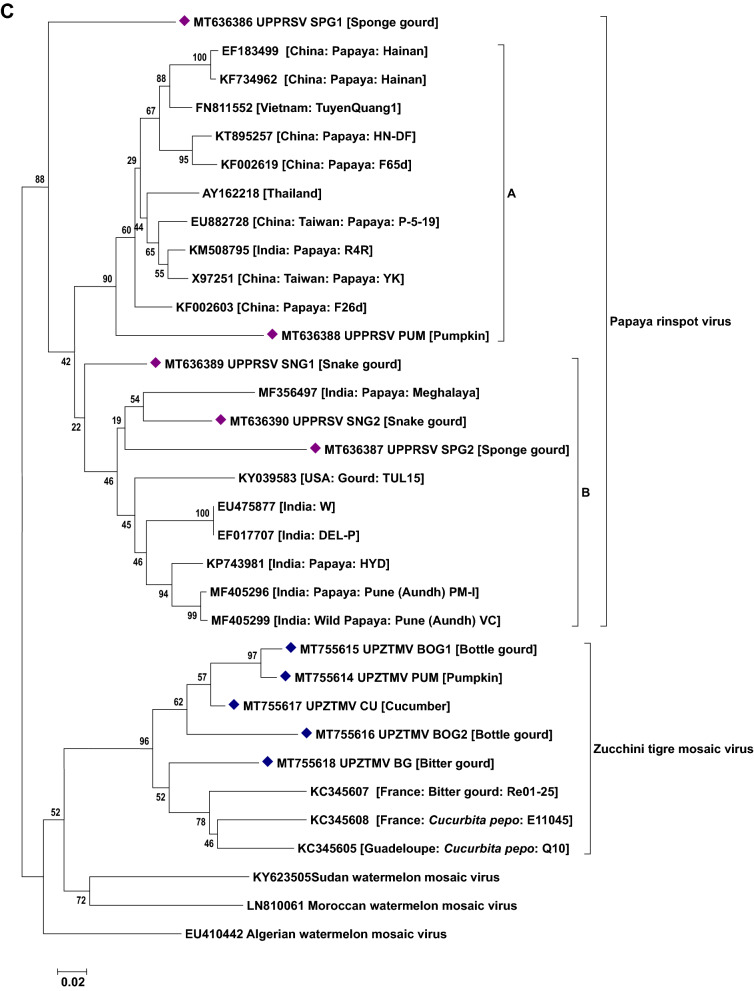

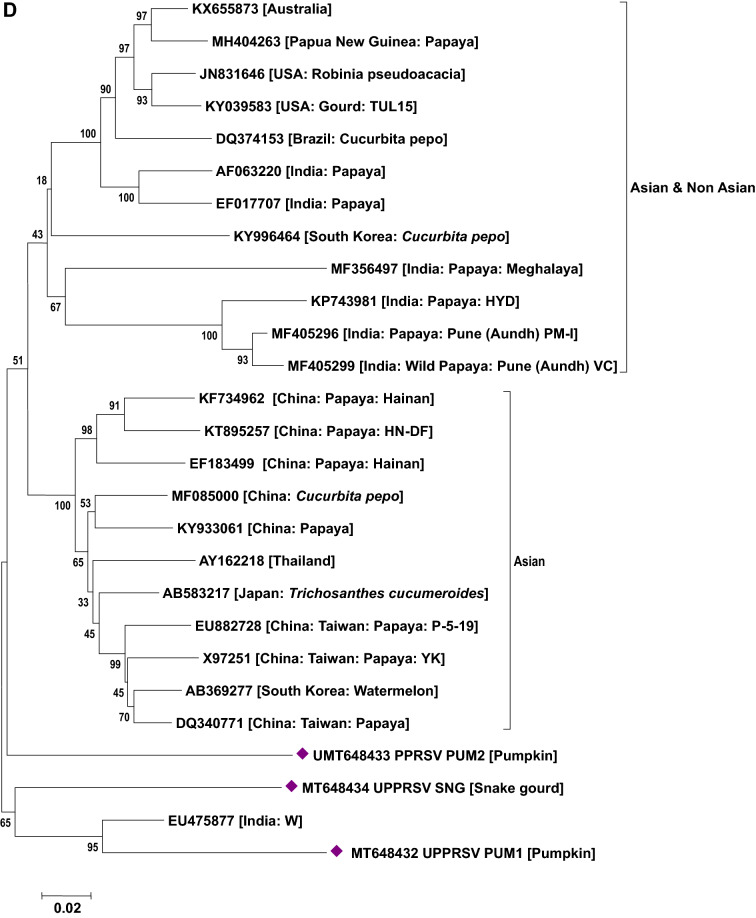

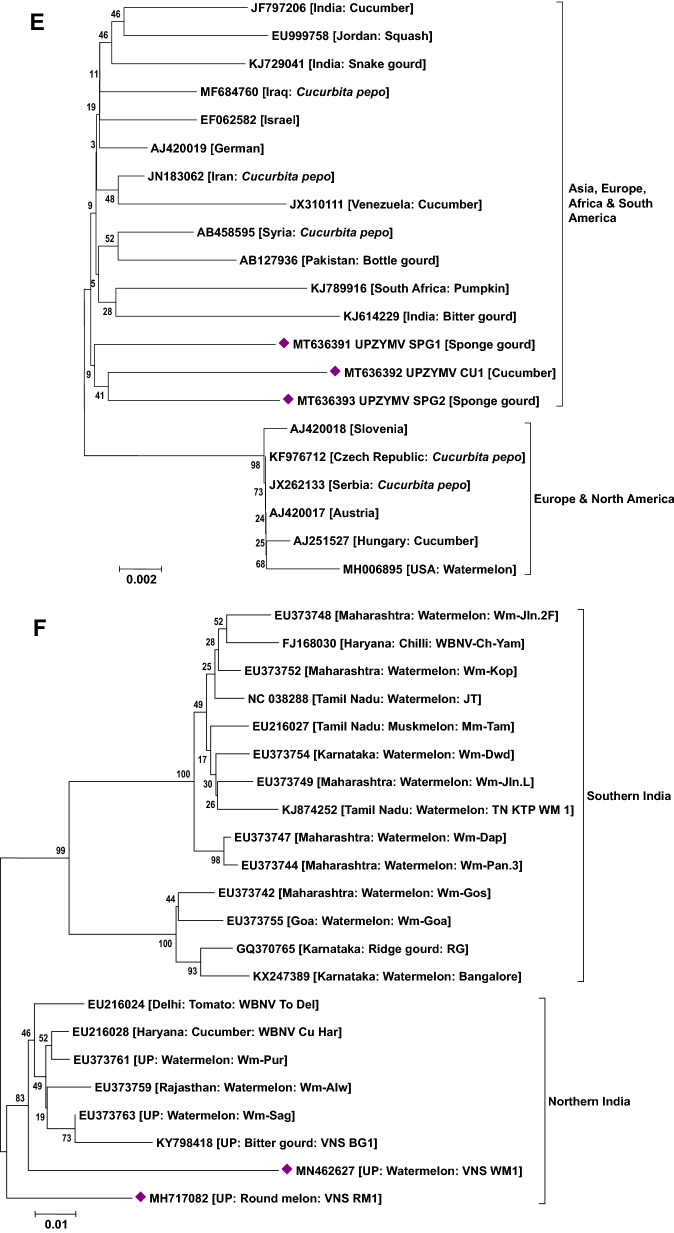

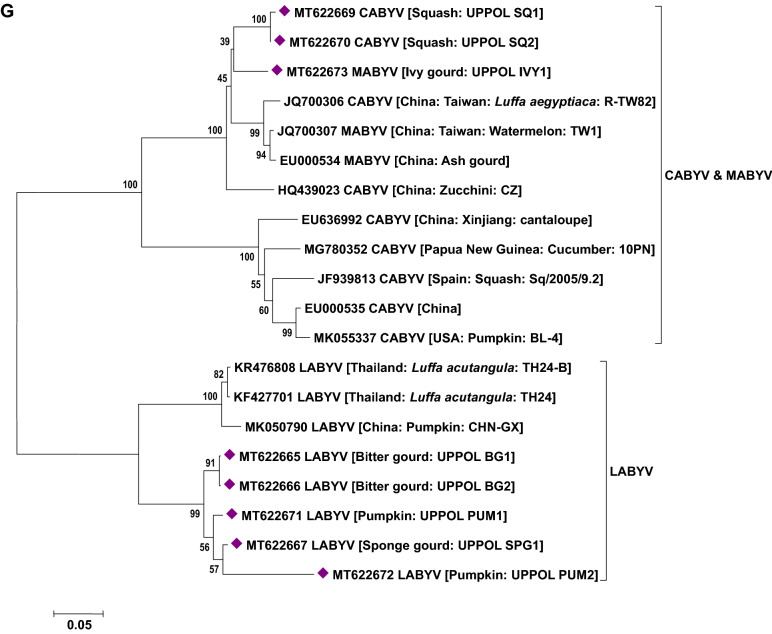
Phylogenetic analysis of (**a**) CMV infecting cucurbits in Uttar Pradesh state with other CMV isolates earlier reported based on 2a region. (**b**) CGMMV infecting cucurbits in Uttar Pradesh state with other CGMMV isolates reported earlier based on coat protein gene. (**c**) Potyviruses (PRSV and ZTMV) infecting cucurbits in Uttar Pradesh state with other earlier reported isolates based on Nib region. (**d**) PRSV infecting cucurbits in Uttar Pradesh state with other PRSV isolates reported earlier based on coat protein gene. (**e**) ZYMV infecting cucurbits in Uttar Pradesh state with other ZYMV isolates reported earlier based on coat protein gene. (**f**) WBNV infecting cucurbits in Uttar Pradesh state with other WBNV isolates reported earlier based on coat protein gene. (**g**) Poleroviruses infecting cucurbits in Uttar Pradesh state with other earlier reported isolates based on coat protein gene. Trees were generated with 1000 bootstrap replications using neighbour joining method in MEGA 7.0 software.

Phylogenetic analysis based on the nucleotide sequence of the 2a fragment showed that a CMV isolate infecting cucurbits shared ancestry with the CMV reported on tomato, snake gourd, bottle gourd, pepper and banana from India (Fig. 5a). Based on the sequence analysis, it has been determined that a CMV strain belonging to serogroup IB is infecting cucurbits in Uttar Pradesh. Similarly, sequence analysis based on the coat protein gene of the CGMMV isolates (Bottle gourd, bitter gourd, sponge gourd, snake gourd, long melon and cucumber) revealed prevalence of two distinct groups in India. The first group forms a cluster with isolates reported from Australia, Greece, China, Korea, Japan and India (clade A) while the second group (clade B) comprises only Indian isolates (Fig. 5b). In the case of PRSV and ZTMV (potyviruses), based on the Nib region, PRSV and ZTMV isolates grouped under two separate clades. Within PRSV isolates, two distinct clades were observed (A and B). One clade shared ancestry with the PRSV strains reported from different countries (clade A) and the second clade (B) clustered particularly with Indian isolates (Fig. 5c). Furthermore, phylogenetic tree generated based on the coat protein region of PRSV formed separate clades from the previously reported isolates from various countries including India (except an Indian PRSV-W strain, EU475877) (Fig. 5d). Being a new virus to India, ZTMV isolates were found to have a close relationship with France isolates (Fig. 5c). With reference to ZYMV, all the study isolates were grouped with isolates reported from Asia, Europe, Africa and South America sharing their common ancestry (Fig. 5e). Similar analysis of WBNV isolates infecting round melon and watermelon typically displayed the same centre of origin with northern India isolates reported earlier and are clearly distinct from the southern India isolates (Fig. 5f). Phylogenetic analysis of polerovirus sequence showed 2 distinct clades of poleroviruses, one comprising CABYV and MABYV and the other comprising LABYV (Fig. 5g).

### Generation of risk map of virus disease incidence using ArcGIS

**Figure 6:**
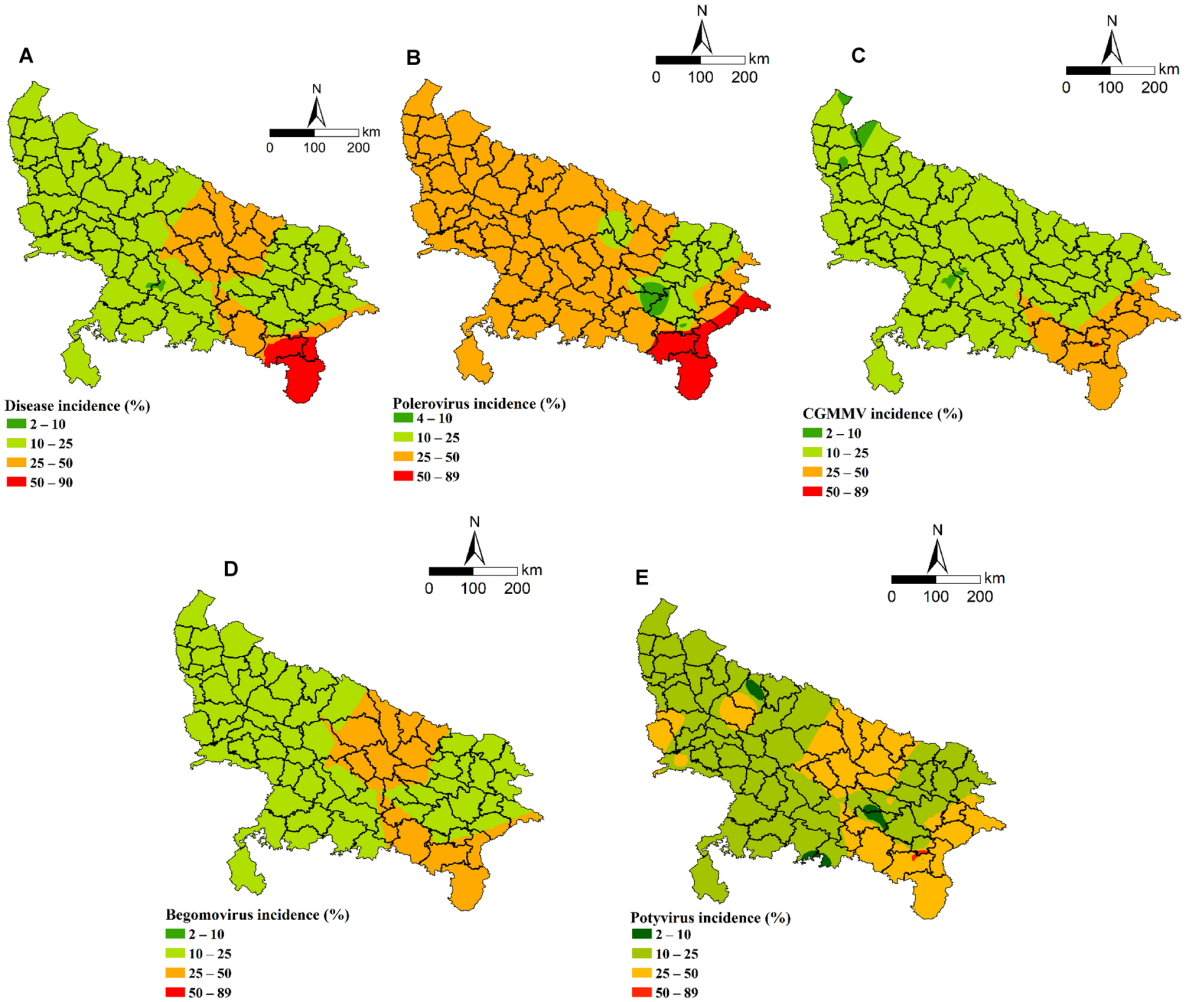
Map showing predicted distribution and incidence proportion of cucurbit viruses in the Uttar Pradesh state using ArcGIS 9.1 software.

Virus distribution and incidence proportion for the four viruses were mapped using the functionalities of ArcGIS 9.1 software and the results are presented in Fig. 6. The maximum area under overall virus disease incidence fall under the category of 10–25% followed by 25–50% (Fig. 6A). The highest incidence was predicted with *Polerovirus* (50–89%) (Fig. 6B) whereas 25–50% incidence for CGMMV (Fig. 6C), *Begomovirus* (Fig. 6D) and *Potyvirus* (Fig. 6E) were observed. Since the survey points for CMV and *Orthotospovirus* are 3 and 2, these viruses were not mapped. The optimum numbers of power values were 7.59, 1, 5.28, 1 and 1 for *Polerovirus*, CGMMV, *Potyvirus, Begomovirus* and overall disease incidence, respectively. The green areas in the interpolated maps indicate very low to low disease incidences while the areas with orange to red colouration indicated moderate to high disease incidences. Around 75% area of Uttar Pradesh state fall under 10–25% disease incidence category for *Begomovirus, Potyvirus* and CGMMV whereas majority area of the polerovirus infection falls under 25–50% disease incidence category (Table 3).

**Table 3:** Percentage of area categorized under each disease incidence category.

Class	Disease incidence range	Potyvirus		Polerovirus		Begomovirus		CGMMV		Overall disease incidence	
		Pixels	% area	Pixels	% area	Pixels	% area	Pixels	% area	Pixels	% area
1	2–10	536	1.68	489	1.53	1	0.00	637	1.99	126	0.39
2	10–25	21,111	66.01	4122	12.89	23,904	74.73	26,454	82.71	23,887	74.66
3	25–50	10,271	32.11	25,043	78.30	8068	25.22	4881	15.26	6518	20.37
4	50–89	65	0.20	2329	7.28	16	0.05	11	0.03	1462	4.57
Total		31,983	100.00	31,983	100.00	31,989	100.00	31,983	100.00	31,993	100.00

## Discussion

In this study, we explored cucurbit fields in different regions of Uttar Pradesh state (UP) (nine agro-climatic zones). Results showed disease incidences in different zones varied significantly. Association of one or more virus species belonging to the genera *Begomovirus, Potyvirus, Cucumovirus, Tobamovirus, Orthotospovirus* and *Polerovirus* were detected. Among the 563 samples tested, 26 samples did not test positive for any tested viruses. These samples may have been infected by viruses other than those tested in this study. In cucurbits, *Begomovirus* infection was the most identified, infecting 523 samples collected from thirteen different cucurbit crops across the UP state. Our result is consistent with previous finding of Nagendran et al.^15^ that Begomoviruses are the most dominant virus (98.6%) in cucurbit samples collected from southern India. High adaptability of *Begomovirus*, polyphagous nature of its vectors and the continuous availability of other host plants plays a major role in the *Begomovirus* infection.

The next to *Begomovirus*, *Potyvirus* was detected among 254 samples collected from 10 cucurbit crops (except watermelon, satputia, musk melon and round melon). Due to the diverse nature of potyviruses prevailing in the cucurbits ecosystem, a single universal primer pair (PNIbF1/PCPR1) could not amplify the majority of samples. Therefore, different sets of primer pairs were used for potyvirus detection (Table 4). Earlier studies from India have documented only the occurrence of PRSV and ZYMV on cucurbits^5,15^. ZYMV isolates from the current study were closely related to previously reported Indian isolates regardless of host, geographic location and year of collection as documented by Chikh-Ali et al.^16^ in Syria. In the present study, in addition to these two potyviruses, occurrence of ZTMV has also been documented for the first time in India. Though diverse potyviruses were infecting cucurbits in India, much diversity was observed only with PRSV, but ZYMV and ZTMV seemed to have low diversity within the species. The widespread distribution of PRSV in single and mixed infections among tested samples indicated that it is an already established virus rather than a recent introduction. In contrast, CGMMV was detected among 223 samples in 11 different crops except for satputia, ridge gourd and round melon. CGMMV isolates in this study showed less diversity irrespective of the host and location of sample collection. Due to its seed transmission nature, single strains of CGMMV might have spread across the country. As different cucurbit crops are cultivated side by side in the Indian subcontinent and harvested manually, mechanical transmission likely plays a major role in the expansion of virus host range.

**Table 4:** Details of primers used in this study.

Primer ID	5′–3′ sequence	Target virus	Target region	Amplicon size (bp)	References
PNIbF1	GGBAAYAATAGTGGNCAACC	Potyvirus	NIB and CP	~ 1100	^17^
PCPR1	GGGGAGGTGCCGTTCTCDATRCACCA				
NIb2F	GTITGYGTIGAYGAYTTYAAYAA		NIB	~ 350	^18^
NIb3R	TCIACIACIGTIGAIGGYTGNCC				
GK PRSV F	GCAATGATAGARTC ATGGGG	PRSV	CP	1264	^15^
GK PRSV R	AAGCGGTGGCGCAGCCACACT				
GK ZYMV F	ATAGCTGAGACA GCACT	ZYMV	CP	1004	^5^
GK ZYMV R2	CGGCAGCRAAACGATAACCT				
CMV 1	GATCATCGCCTGAGAATA	CMV	2a	~ 400	^19^
CMV 2	TTCCAGAGATGCCTTCG				
gL3637	CCTTTAACAGTDGAAACAT	*Orthotospovirus*	RdRp	~ 800	^20^
gL 4435c	CATDGCRCAAGARTGRTARACAGA				
GK PBNV CP F	ATGTCTAACGTYAAGCAGCTC	GBNV	CP	~ 900	^21^
GK PBNV CP R	TTACAATTCCAGCGAAGGAC				
GK WBNV CP F	AATAAACTAATGACACACACAAA	WBNV	CP	~ 950	^22^
GK WBNV CP R	ACGTTTCCAKAGTAAACACCAT				
GK CGMMV F	TAAG CGGCATTCTAAACCTCCA	CGMMV	CP	604	^6^
GK CGMMV R	CACTATGCACTTTG GTGTGC				
PolGenUp2	GATGARGGTCGYTACCG	Polerovirus	CP	593	^23^
PolGenDown2	ACCTCGACTTTRAARCC				
Potex 2RC	AGCATRGCNSCRTCYTG	Potexvirus		~ 600	^24^
Potex 5	CAYCARCARGCMAARGAYGA				
CriniRdRp251F	TNGGNAARGGNGARAG	Crinivirus	RdRp	~ 800	^25^
CriniRdRp 995R	GTRTTNGAYAACCAHGTRTTHG				
PAL1c1960	ACNGGNAARACNATGTGGGC	Begomovirus	AC1 and AC2	~ 1200	^26^
PAR1v722	GGNAARATHTGGATGGA				

Besides, CMV was the least distributed virus detected only among 12 samples of bitter gourd, sponge gourd and ridge gourd. Though CMV has wide host range, the reason for its limited distribution among cucurbits remains unclear. Similarly, limited occurrence of CMV in cucurbits from commercial growers among the several viruses tested has been documented earlier^15,27^. Despite the fact that *Orthotospovirus* has been well documented on various cucurbits in the Indian subcontinent, only 10 samples of round melon and watermelon crops were found to be infected with *Orthotospovirus* in the UP state. There have been several reports on the occurrence of *Orthotospovirus* in southern India but not from northern India. Recently, research from our laboratory documented for the first time in India *Orthotospovirus* infection on cucurbits including PBNV on bitter gourd^20^ and WBNV on round melon^28^. This study further reinforces the previous findings of *Orthotospovirus* being an emerging threat to the cultivation of vegetable crops in northern India. Three species of cucurbit infecting poleroviruses (CABYV, MABYV and LABYV) were identified and characterized in pumpkin, bitter gourd, sponge gourd, squash and ivy gourd. A previous study from southern India has documented the infection of bitter gourd and teasel gourd by CABYV^29,30^. Findings from the present study further confirm the existence of CABYV on cucurbits cultivated in northern India with an expanded host range, and the existence of other species (MABYV and LABYV) may be indicative of its emergence in India.

Further our study shows the predominance of mixed infection (64%) over single infection (32%) in infected samples. Although up to 32% single infection due to Begomoviruses, causing mosaic and mottling symptoms was observed in cucurbit samples, double infection by *Begomovirus* coupled with *Potyvirus/ Tobamovirus/ Polerovirus* (44%) was by far the most common in tested samples. Infection of cucurbits by triple viruses (*Begomovirus* + *Potyvirus* + *Tobamovirus*) was recorded in 16% of the total tested samples. Similar results have been observed in India and Oklahoma with preponderance of double infection over triple infection^15,27^. Additionally, Kone et al.^31^ observed average multiple virus infections among 15% of cucurbits samples in Cote d’Ivoire. Interestingly, mixed infection of cucurbits by four viruses (*Begomovirus* + *Potyvirus* + *Tobamovirus* + *Polerovirus*) was observed in very few samples as previously reported on squash, melon and cucumber in Iran^32^.

The main aim of risk mapping of ArcGIS is to find the hazard level of the virus which is causing threat to crop cultivation^33,34^. It provides map showing probability of the occurrence of disease in the unexplored area based on the data collected from few pockets of the state. This gives clarity about the hot spot and cold spot for each virus separately as well as for the overall virus diseases infecting cucurbits. To our knowledge, this is the first of its kind in generating the risk map for virus diseases infecting cucurbits.

Overall, the present work outlined the current status of cucurbits viruses in UP state and provided conclusive evidence of viral diversity and the potential presence of new viruses currently emerging in this area. The distribution pattern of different cucurbit viruses in this region will help to identify the hot spots for viruses and will facilitate to devise efficient and eco-friendly integrated management strategies for management. Additionally, we identified many new viruses such as CMV on sponge gourd, bitter gourd and ridge gourd; CGMMV on long melon; *Polerovirus* on squash, pumpkin, ivy gourd and sponge gourd; and ZTMV on pumpkin, bottle gourd, bitter gourd, squash and cucumber. Further studies are needed to explore and characterize new and unidentified viruses which have been reported from different parts of world.

## Materials and methods

### Sample collection and analysis

An intensive study was conducted on cucurbitaceous crops during the cropping seasons in 2018 from all nine agro-climatic zones of Uttar Pradesh state, India (Fig. 2). Around 200 farmers’ fields were visited during the sampling and a total of 563 samples from 14 different cucurbit crops suspected to have virus infection were collected by following relevant guidelines and permission from respective farmers for analysis (Supplementary Table 1; Supplementary Fig. 2). Percent disease incidence per field was estimated based on the number of plants showing symptoms and the total number of plants observed.

### Detection of begomovirus

Total DNA was extracted from leaf tissues (100 mg) of both symptomatic and non-symptomatic plants using the cetyl trimethyl ammonium bromide (CTAB) method as described by Doyle and Doyle^35^ and subjected to PCR amplification using universal Begomovirus primer pair PAL1c1960/PAR1v722 (Table 4).

### Detection of RNA viruses

Total RNA was extracted from 100 mg of each symptomatic and apparently healthy leaf samples using TriReagent (Sigma Aldrich, USA). Total RNA was subjected to reverse-transcription polymerase chain reaction (RT-PCR) for the preparation of cDNA using the RevertAid First Strand cDNA synthesis kit (Thermo Scientific, USA) according to the manufacturer's instructions. Prepared cDNA was used for virus detection through PCR amplification using specific primers (Table 4).

### Cloning, sequencing and sequence analysis

Amplified PCR products of representative samples were purified using the QIAquick Gel Extraction Kit (Qiagen) and cloned in the pGEM-T Easy Vector System (Promega Corp.)^36^. Plasmid DNA preparations were obtained using Wizard Plus Minipreps DNA Purification (Promega Corp). Two clones were selected from each sample for sequencing and sequencing was performed at the Delhi University-South campus, New Delhi (India). Sequences were analyzed using the Basic Local Alignment Search Tool (BLAST) for the identification of virus at species level^37^. The top three to five hits against each database were included in the analysis for each sequence. The molecular evolutionary genetics analysis software (MEGA, version 7) was employed to determine the phylogenetic relationship of the study isolates with the earlier reported viruses using the neighbour joining method^38^.

### Application of ArcGIS for disease mapping

The GPS (geographical positioning system) data and disease incidence data of the explored areas were collected to be used for mapping the epidemiological distribution and incidence proportion of viruses infecting cucurbit crops. The spatial variability maps of *Polerovirus*, CGMMV, *Potyvirus*, *Begomovirus* and overall disease incidence in nine agro-climatic zones were prepared using Geostatistical Analyst extension in ArcGIS 9.1 software. Inverse distance weighted (IDW) method was used for interpolation. The IDW is a simple interpolation technique which is based on the assumption that the variable values at unmeasured locations are influenced most by nearby observation points and less by distant points. This technique assumes that each measured location has a local effect, which reduces with distance by means of the utilization of a power parameter^39^. The formula of IDW^40^ is given as follows:

$${\hat{\text{Z}}}\left( {{\text{S}}_{0} } \right) = { }\mathop \sum \limits_{{{\text{i}} = 1}}^{{\text{n}}} {\uplambda }_{{\text{i}}} {\text{Z}}\left( {{\text{S}}_{{\text{i}}} } \right)$$

where ${\hat{\text{Z}}}$(S_0_) is the predicted value at location S_0_, n is the number of measured sample points surrounding the prediction location, λ_i_ is the weight assigned to each measured point, Z(S_i_) is the observed value at the location S_i_.

The formula to calculate the weights is given as follows:

$${\uplambda }_{{\text{i}}} = { }\frac{{{\text{d}}_{{{\text{i}}0}}^{{ - {\text{p}}}} }}{{\mathop \sum \nolimits_{{{\text{i}} = 1}}^{{\text{n}}} {\text{d}}_{{{\text{i}}0}}^{{ - {\text{p}}}} }}$$

p is power parameter which reduces with increasing distance, d_i0_ is the distance between the prediction location (S_0_) and each of the measured locations (S_i_).

The optimal power value of IDW was estimated using root mean square error of cross-validation (RMSECV). Power value with the lowest RMSECV was selected for IDW interpolation^41^. A variable search radius with maximum of 15 sample points and minimum of 10 sample points was used.

## Supplementary Information

Supplementary Table 1.

Supplementary Figures.
