# Author Correction: Investigations on annual spreading of viruses infecting cucurbit crops in Uttar Pradesh State, India

**DOI:** 10.1038/s41598-021-00750-4

**Published:** 2021-11-09

**Authors:** Shweta Kumari, Nagendran Krishnan, Vikas Dubey, Bappa Das, Koshlendra Kumar Pandey, Jagdish Singh

**Affiliations:** 1 ICAR-Indian Institute of Vegetable Research, Varanasi, Uttar Pradesh 221305 India; 2 ICAR-Central Coastal Agricultural Research Institute, Old Goa, Goa 403402 India

Correction to: *Scientific Reports* 10.1038/s41598-021-97232-4, published online 09 September 2021

The Acknowledgements section in the original version of this Article was incomplete.

“Authors are thankful to Dr. Debashis Chakraborty, ICAR-IARI, New Delhi for facilitating us in QGIS data analysis and Dr. Stephen Wegulo, University of Nebraska-Lincoln for helping us in editing the manuscript. Further authors are very much thankful to Director, ICAR-IIVR, Varanasi for facilitating to conducting the research.”

now reads:

“Authors are thankful to Dr. Debashis Chakraborty, ICAR-IARI, New Delhi for facilitating us in QGIS data analysis and Dr. Stephen Wegulo, University of Nebraska-Lincoln for helping us in editing the manuscript. Further authors are very much thankful to the Director, ICAR-IIVR, Varanasi for facilitating to conducting the research and for funding publication charges.”

The original Article has been corrected.

