# Supplementary material for: Investigations on annual spreading of viruses infecting cucurbit crops in Uttar Pradesh State, India
Source: Sci Rep. 2021 Sep 9;11:17883. doi: 10.1038/s41598-021-97232-4 (PMC8429706; doi:10.1038/s41598-021-97232-4)
Supplement: Supplementary file 1 — Supplementary Table 1. [file 41598_2021_97232_MOESM1_ESM.docx]

**Supplementary Table 1. List of 14 cucurbitaceous crops surveyed for viral diseases in the Uttar Pradesh State, India**

| **S. No.** | **Common Name** | **Botanical Name** |
| --- | --- | --- |
|  | Bitter gourd | *Momordica charantia* L. |
|  | Bottle gourd | *Lagenaria siceraria* (Molina) Standl) |
|  | Cucumber | *Cucumis sativus* L |
|  | Ivy gourd | *Coccinia grandis* (L. |
|  | Long melon | *Cucumis melo*var. *utilissimus* (Duthic and Fuller) |
|  | Musk melon | *Cucumis melo* L. |
|  | Pumpkin | *Cucurbita maxima* Duchesne |
|  | Ridge gourd | *Luffa acutangula* Mill. |
|  | Round melon | *Praecitrullus fistulosus* |
|  | Satputia | *Luffa hermaphrodita* |
|  | Snake gourd | *Trichosanthes cucumerina* L. |
|  | Sponge gourd | *Luffa aegyptiaca* |
|  | Squash | *Cucurbita pepo* |
|  | Watermelon | *Citrullus lanatus* (Thunb.) |
