# Supplementary material for: Investigations on annual spreading of viruses infecting cucurbit crops in Uttar Pradesh State, India
Source: Sci Rep. 2021 Sep 9;11:17883. doi: 10.1038/s41598-021-97232-4 (PMC8429706; doi:10.1038/s41598-021-97232-4)
Supplement: Supplementary file 2 — Supplementary Figures. [file 41598_2021_97232_MOESM2_ESM.pptx]

## Slide 1
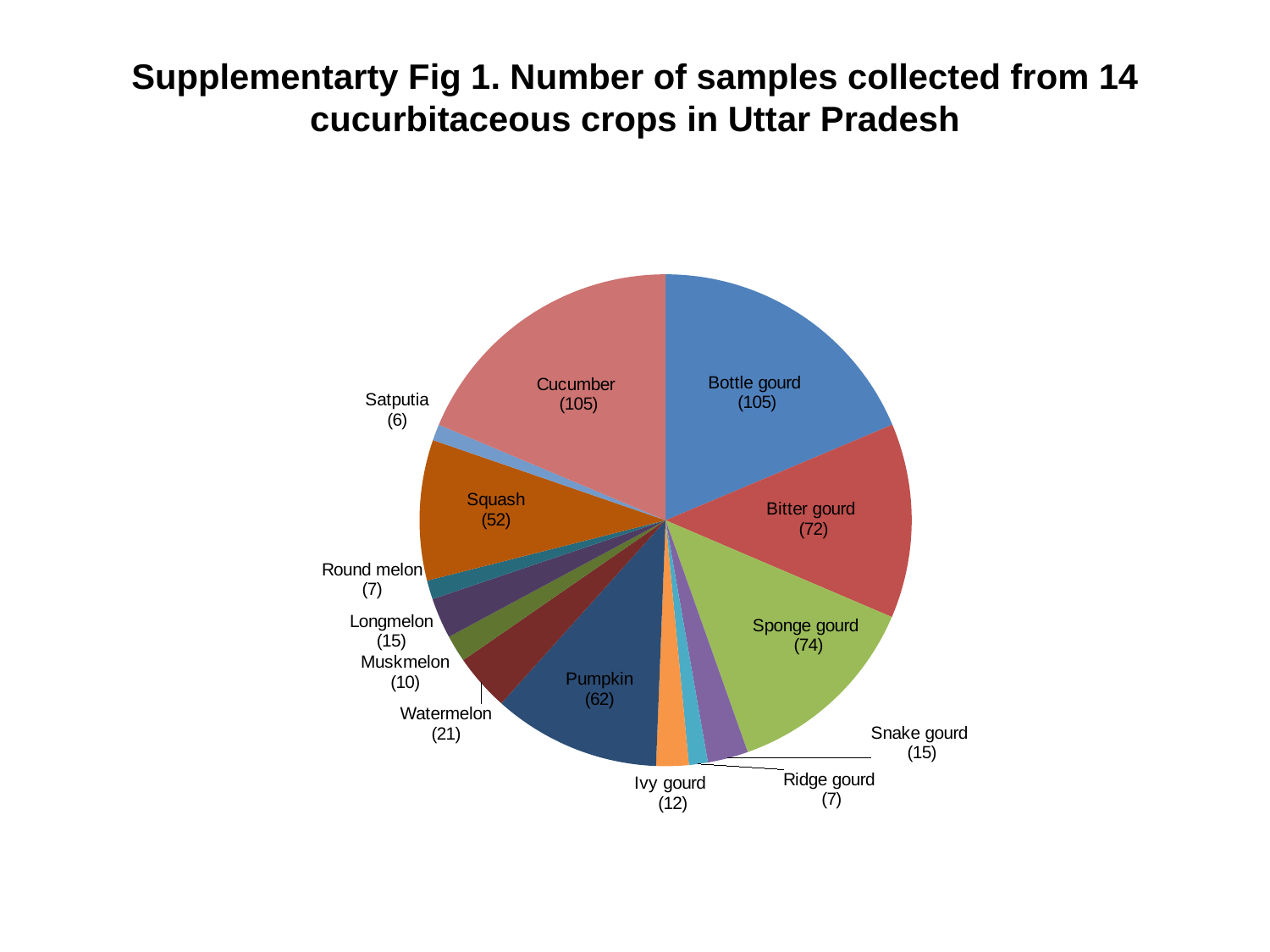

# Supplementarty Fig 1. Number of samples collected from 14 cucurbitaceous crops in Uttar Pradesh
### Chart
| Category | |
|---|---|
| Bottle gourd | 105.0 |
| Bitter gourd | 72.0 |
| Sponge gourd | 74.0 |
| Snake gourd | 15.0 |
| Ridge gourd | 7.0 |
| Ivy gourd | 12.0 |
| Pumpkin | 62.0 |
| Watermelon | 21.0 |
| Muskmelon | 10.0 |
| Longmelon | 15.0 |
| Round melon | 7.0 |
| Squash | 52.0 |
| Satputia | 6.0 |
| Cucumber | 105.0 |

## Slide 2
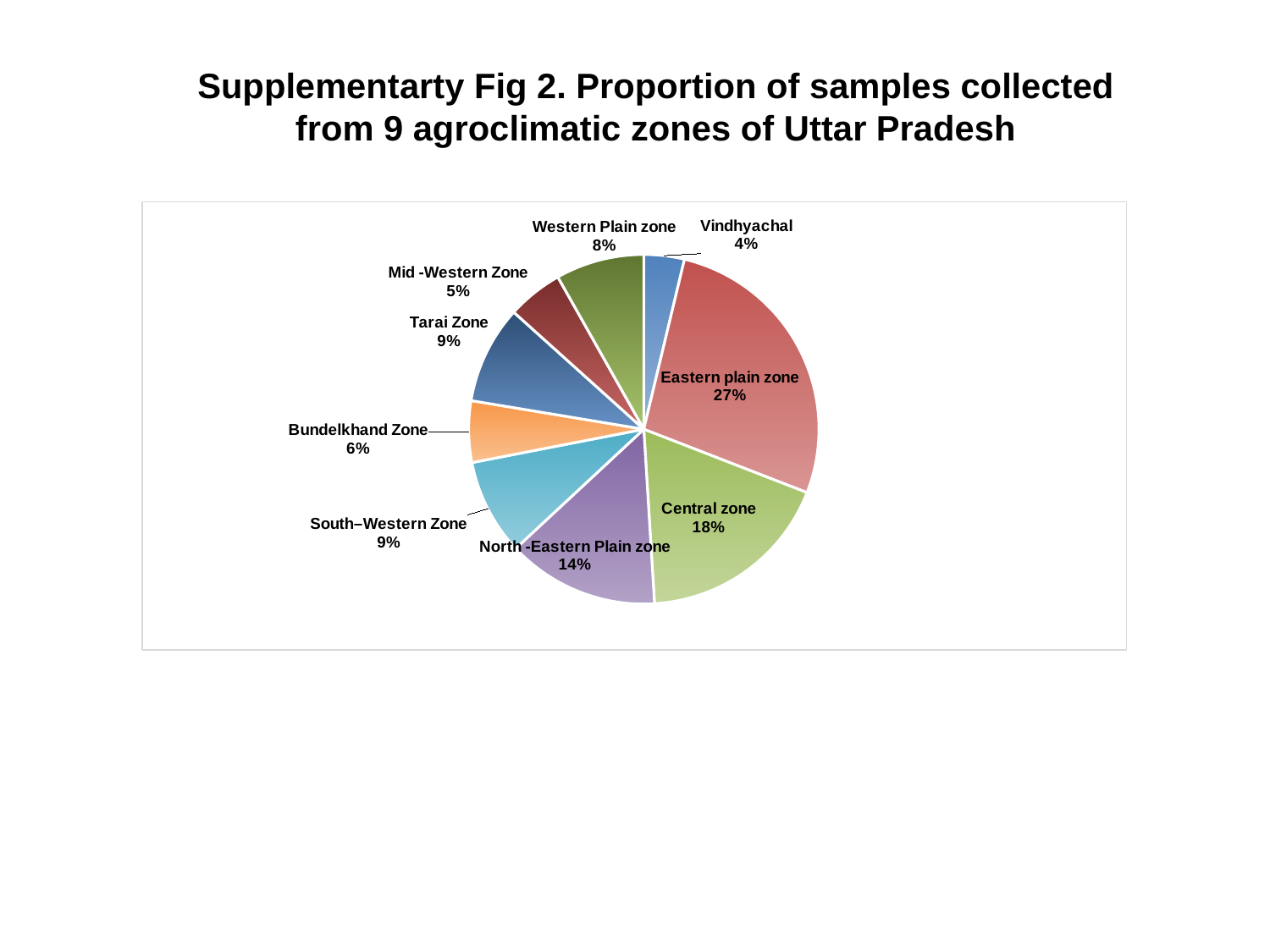

Supplementarty Fig 2. Proportion of samples collected from 9 agroclimatic zones of Uttar Pradesh
### Chart
| Category | |
|---|---|
| Vindhyachal | 21.0 |
| Eastern plain zone | 153.0 |
| Central zone | 102.0 |
| North -Eastern Plain zone | 79.0 |
| South–Western Zone | 50.0 |
| Bundelkhand Zone | 32.0 |
| Tarai Zone | 51.0 |
| Mid -Western Zone | 29.0 |
| Western Plain zone | 46.0 |

## Slide 3
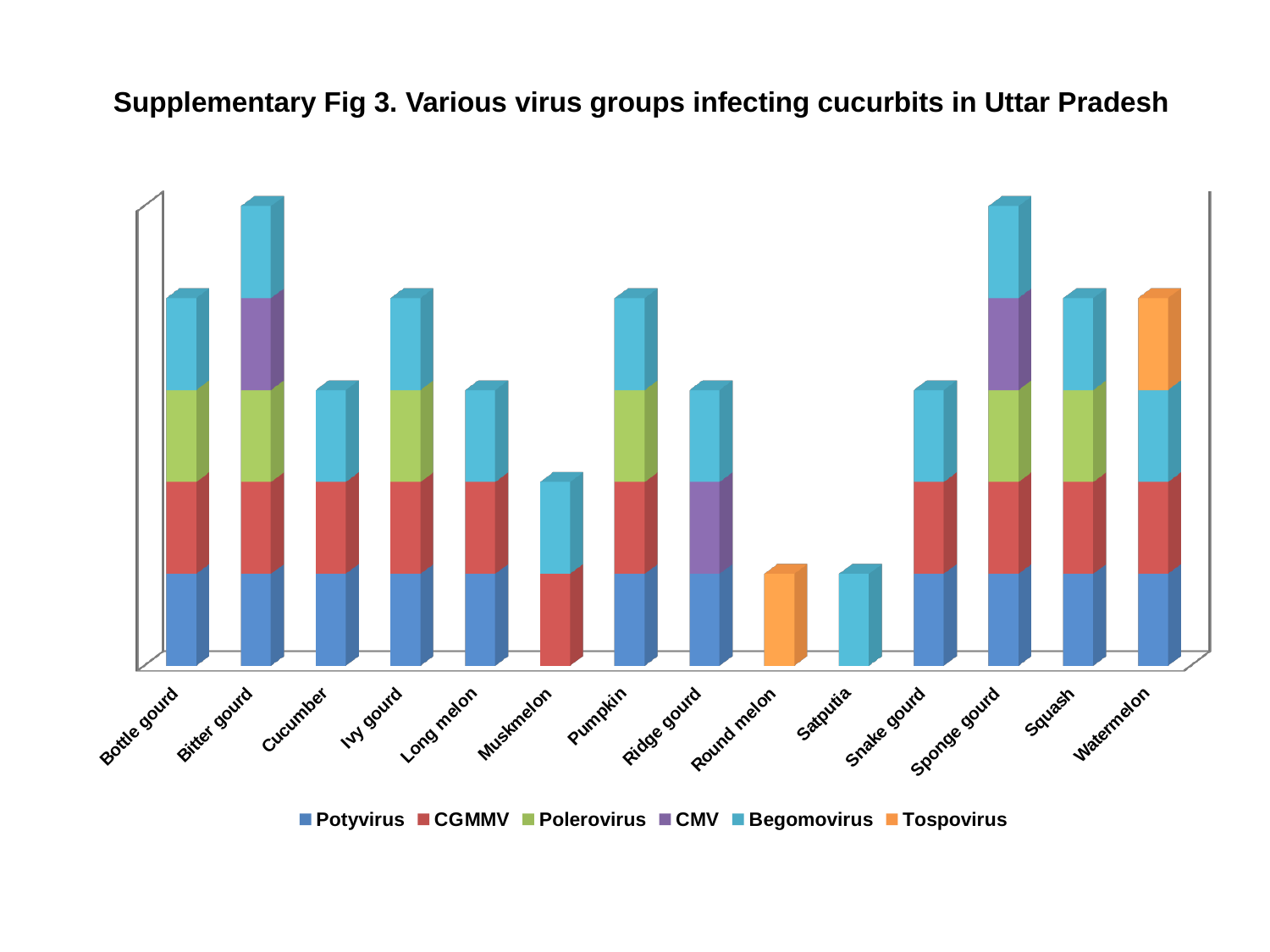

# Supplementary Fig 3. Various virus groups infecting cucurbits in Uttar Pradesh
[unsupported chart]
